# A Mini Review Focused on the Recent Applications of Graphene Oxide in Stem Cell Growth and Differentiation

**DOI:** 10.3390/nano8090736

**Published:** 2018-09-18

**Authors:** Alexander Halim, Qing Luo, Yang Ju, Guanbin Song

**Affiliations:** 1 Key Laboratory of Biorheological Science & Technology, Ministry of Education, College of Bioengineering, Chongqing University, Chongqing 400030, China; 20161500175@cqu.edu.cn (A.H.); qing.luo@cqu.edu.cn (Q.L.); 2 Department of Mechanical Science and Engineering, Nagoya University, Nagoya 464-8603, Japan; ju@mech.nagoya-u.ac.jp

**Keywords:** graphene oxide, stem cells, growth, cell differentiation, biomaterials

## Abstract

Stem cells are undifferentiated cells that can give rise to any types of cells in our body. Hence, they have been utilized for various applications, such as drug testing and disease modeling. However, for the successful of those applications, the survival and differentiation of stem cells into specialized lineages should be well controlled. Growth factors and chemical agents are the most common signals to promote the proliferation and differentiation of stem cells. However, those approaches holds several drawbacks such as the negative side effects, degradation or denaturation, and expensive. To address such limitations, nanomaterials have been recently used as a better approach for controlling stem cells behaviors. Graphene oxide is the derivative of graphene, the first two-dimensional (2D) materials in the world. Recently, due to its extraordinary properties and great biological effects on stem cells, many scientists around the world have utilized graphene oxide to enhance the differentiation potential of stem cells. In this mini review, we highlight the key advances about the effects of graphene oxide on controlling stem cell growth and various types of stem cell differentiation. We also discuss the possible molecular mechanisms of graphene oxide in controlling stem cell growth and differentiation.

## 1. Introduction

The potential use of stem cells has attracted much attention due to their unique ability to self-renew and differentiate into multiple types of cells. Therefore, stem cells have been utilized for various applications, such as disease modeling, drug discovery and testing, regenerative therapy, and tissue engineering [1,2,3,4] However, for the successful of stem cells-based application, the differentiation of stem cells into specialized cells should be well controlled. Conventionally, biochemical signals, including growth factors and chemical agents, are commonly used to expand and control the differentiation of stem cells. However, the stimulation of stem cell differentiation by using growth factors and chemical agents are unstable, inefficient, and hazardous [5,6,7,8]. To address these limitations, nanomaterials have been recently used to control stem cell growth and differentiation. 

Latterly, graphene (Gp), a two-dimensional (2D) carbon-based nanomaterials containing a single layer of carbon atoms packed in a honeycomb crystal lattice with sp2 hybridization, and its derivatives, graphene oxide (GO), and reduced graphene oxide (rGO) have attracted many scientific fields due to their extraordinary properties, including high surface area, remarkable electrical and thermal conductivities, strong mechanical strength, and optical transparency [9,10,11,12,13]. Moreover, they have been shown to influence the self-renewal and differentiation of stem cells. GO is the highly oxidized form of Gp, which has several functional groups (e.g., hydroxyl, carboxyl, and epoxy groups). Because of those functional groups, GO can be easily combined with other biomolecules and biomaterials. Moreover, the advantage of GO as compared to Gp is its ease of dissolving in water and other organic solvents, due to the polar oxygen functional groups. Several reports have demonstrated that GO is less cytotoxic than graphene and other derivatives due to its surface functionalization. The oxygen content of GO provides the hydrophilic characteristic that enables it to avoid agglomeration in cell culture medium. The agglomeration phenomenon would limits the nutrient supply and subsequently induces oxidative stress, which triggers apoptotic pathways [14]. Moreover, the oxygen functional groups of GO could control the extracellular matrix (ECM) protein adsorption that further lead to cell adhesion and proliferation enhancement [15,16]. On the other hand, another derivative of Gp, rGO, can be produced by removing most of the oxygen-containing groups of GO resulting in the recovery of its electrical conductivity properties that has been shown to enhance neurogenesis [17]. In addition, the sharp edges and oxygen-functional groups of GO could induce the bacterial cell membrane disruption and oxidative stress [18,19,20], which further lead to the enhancement of angiogenesis and osteogenesis [21,22]. The antibacterial and antimicrobial properties of GO could make it a promising material for tissue regeneration application.

In this mini review, we summarize the recent progress in the potential application of GO for regulating stem cell behavior. We first outline the effect of GO in stem cell growth and proliferation. Subsequently, we highlight the influence of GO in stem cell differentiation. Finally, we consider some molecular mechanisms that underlie the interaction between GO and stem cells, with the hope that such an understanding will enable the optimization of GO to improve the clinical outcomes.

## 2. Effect of Graphene Oxide on Stem Cell Growth and Proliferation

Along with the increasing interest of using GO-based nanomaterials for stem cell applications, a number of studies have endeavored to analyze its toxicity and biocompatibility. There are several major parameters that need to be taken into consideration in order to define the degree of biodegradability and biocompatibility of overall nanomaterials, including their size, shape, concentration and time of incubation, and surface area design or functionalization. Moreover, the cytotoxicity effect of GO is also different to each other, depending on the type of stem cell (Table 1).

Wei et al. showed that GO at 0.1 µg/mL concentration could significantly promote the proliferation of bone marrow-derived mesenchymal stem cells (BMSCs). However, the elevation in GO concentration up to 1 and 10 µg/mL would lead to the reduction of cell proliferation. Moreover, the cells’ size become smaller and shrinkage occurs at concentration of ≥1 µg/mL [23]. Besides the concentration effect, the size and shape of GO, as well as its exposure time, have also been investigated. Several reports have demonstrated that the smaller size of GO have higher cytotoxicity. Akhavan et al. reported that, even at low concentration (1 µg/mL), rGO with the small lateral size is sufficient enough to induce cytotoxicity after a short period of time exposure. On the other hand, the large size of rGO exhibited its cytotoxicity only at high concentration (≥100 µg/mL) and after long period of time exposure [24]. In another study, the authors have also investigated the effect of GO shape on stem cell cytotoxicity. Their results showed that the single layer rGO nanoribbons were shown to cause much higher cell destruction than the rGO sheets under the same conditions.

Furthermore, more and more researchers have intensively modified the GO surface structure in order to improve and optimize its biological effects on stem cells. Due to its oxygen functional groups, GO could be easily functionalized with other materials. For example, the functionalization of GO with polyethylene glycol (PEG) could enhance the aqueous stability of GO and further elevate the proliferation rates of MSCs. Kumar et al. showed that the addition of amine groups on GO surface could elevates the proliferation rate of MSCs due to the synergistic effects of oxygen-containing and amine groups, which could lead to the higher adsorption of cell-adhesive proteins [22].

## 3. Effect of Graphene Oxide on Stem Cell Differentiation

### 3.1. Effect on Embryonic Stem Cell Differentiation

Embryonic Stem Cells (ESCs) are pluripotent cells that have the capability to differentiate into all of cell types in three embryonic germ layers, including ectoderm, endoderm, and mesoderm lineages [30]. Due to the unlimited proliferation and differentiation capacities, ESCs have been widely used in tissue engineering and regenerative medicine field. Recently, GO have been reported to promote mouse ESC differentiation towards dopamine neurons and hematopoietic lines [31,32]. Garcia-Alegria et al. have investigated the effects of GO on promoting ESC differentiation towards haematopoietic lineages. Their results showed that GO coated coverslips substrates could significantly enhance the differentiation of both murine and human ESCs into haematopoietic progenitor cells [31].

Yang et al. studied the effects of carbon nanotubes (CNTs), graphene (Gp), and graphene oxide (GO) on the dopamine neural differentiation of mouse ESCs. Their results showed that only GO could effectively promoted the dopamine neural differentiation of ESCs by comparing the dopamine neuron-related gene expression with the control (without nanoparticles) and two other nanoparticles groups (CNTs and Gp). In addition, the effect of GO on promoting ESC differentiation towards dopamine neuron cells is dose dependent and significantly affects the dopamine-related gene expression. The authors used a dose of 1, 20, 50, and 100 mg/mL GO and showed that GO at concentration 100 mg/mL have threefold elevation of GFP-positive dopamine neurons. While exposure with concentration of 1 mg/mL GO did not show any elevation when compared with control, CNTs, and Gp groups. Moreover, the 100 mg/mL concentration could also enhance the Tyrosine hydroxylase (TH) expression by threefold [32].

### 3.2. Effect on Induced Pluripotent Stem Cell Differentiation

Induced pluripotent stem cells (iPSCs) are somatic cells that were reprogrammed to the pluripotent state by using overexpression of four transcription factors (Oct4, Klf4, Sox2, and c-Myc). iPSCs have similar capacity as embryonic stem cells to differentiate into almost all cell types in three germ layers [33]. Moreover, the use of iPSCs could overcome the ethical issue of using ESCs and allow the creation of cell lines that are genetically tailored to a specific patient. Therefore, iPSCs could offer novel opportunities for tissue engineering and regenerative medicine. Nevertheless, the current directed differentiation protocols by using biochemical agents is inefficient and it requires complex cultures protocols.

Recently, a study that showed GO could affect the differentiation ability of iPSCs, especially into endodermal lineage has been published. Chen et al. used Gp and GO as a 2D substrate to investigate their effects on iPSC differentiation. Interestingly, their results showed that Gp and GO-coated substrate have different effects on iPSC differentiation. The differentiation of iPSCs were inhibited on Gp group. However, the iPSC differentiation was significantly enhanced in the GO group, especially towards endodermal lineage. The pluripotency markers, Nanog and Oct4, were significantly downregulated compared to the control and Gp groups. While, the expressions of Gata4 and Ihh (endodermal markers) were significantly upregulated at day 9, the authors speculated that the surface groups of GO that bind to the surface receptor of iPSCs might be the cause of the spontaneous differentiation of iPSCs [34].

### 3.3. Effect on Mesenchymal Stem Cell Differentiation

Mesenchymal stem cells (MSCs) are multipotent cells that have the potential to self-renew and differentiate into a number of specialized lineages, including osteoblasts, adipocytes, myoblasts, chondroblasts, tenocytes, and neurons [35]. Moreover, MSCs have immunomodulatory properties that enable them to avoid the immune rejection after transplantation [36]. Due to those abilities, MSCs are considered to be an attractive candidate for tissue engineering and regenerative medicine.

Several studies have found that GO has the ability to control the differentiation of MSCs towards osteoblasts, adipocytes, chondroblasts, and neurons. Wei et al. found that pristine GO nanosheets could promote the osteogenic differentiation of bone marrow mesenchymal stem cells (BMSCs). The authors used several different concentrations and found that GO at concentration 0.1 µg/mL was the optimum concentration for promoting osteogenic differentiation. Their research conclusively showed that the osteogenesis effect of GO was dose-dependent and through the activation of Wnt/β-catenin signaling pathway [23]. Moreover, the use of GO could significantly reduce the dosage of bone morphogenic protein-2 (BMP-2) that is required for promoting osteogenic differentiation. BMP-2 is a potent inducer for promoting osteogenesis, and it is clinically used to regenerate bone. However, high doses of BMP-2 could lead to several undesirable adverse effects and it could make the treatment cost become more expensive [37].

Interestingly, GO could be used to promote osteogenic differentiation of MSCs without the help from osteogenic induction supplements such as, dexamethasone, β-glycerolphosphate, and ascorbic acid [38]. Tang et al. modified the structure of GO by functionalizing it with methacrylate groups to form acrylated GO (GO-ac). The GO-ac provided nanotopographical cues that could promote the ostegenic differentiation of MSCs without any osteogenic induction supplements. Furthermore, due to the outstanding mechanical properties and easy functionalization of GO with other biomaterials, GO could be combined with collagen-based scaffolds, which enhance the stiffness of the scaffolds up to threefold and subsequently promote the osteogenic differentiation of MSCs [39].

Other than being able to promote osteogenic differentiation, GO could also promote the adipogenic differentiation of MSCs. Lee et al. compared the effects of graphene and GO on promoting MSC differentiation towards osteoblast and adipocytes. Their results showed that both graphene and GO could promote osteogenic differentiation. However, the situation is different when MSCs were induced to differentiate towards adipocytes on graphene and GO. The results showed that graphene inhibited the adipogenesis, but on the other hand, GO strongly enhanced the adipogenesis. According to the results, GO has high affinity towards insulin, which is the main inducer of adipogenesis. Hence, adipogenesis could be enhanced [40]. However, different from the osteogenesis phenomenon, the GO substrate alone could not induce the adipogenic differentiation of MSCs without the help from adipogenic chemical inducers. Therefore, the authors conclude that the adipogenesis enhancement it is not due to the substrate nanotopography. This study is supported by Patel et al. who conducted a study about guiding adipogenic differentiation of tonsil-derived MSCs by using composite system of GO and polypeptide thermogel (GO/P). Their studies proved that high amount of insulin could be adsorbed on GO surface and formed a multilayer of insulin that further enhances the adipogenic differentiation of MSCs. This interaction could occur due to the polar surface functional groups, including hydroxyl and carboxylate. The potential use of GO on adipogenic differentiation is very useful in practical application, like reconstructive surgery [41].

Articular cartilage is a tissue that has very low regenerative ability due to the low cell density and abundant ECM [42]. Therefore, articular cartilage repair and regeneration become a serious problem worldwide. The appropriate biomimetic scaffolds and chondrocytes are required for cartilage repair and regeneration. Recently, the use of GO-based scaffolds has been explored to guide the chondrogenic differentiation of MSCs. Lee et al. used GO as a soluble and three-dimensional scaffolds to support pellet formation and differentiation of human MSCs (hMSCs) towards chondrogenic lineages because the 2D-based cultures would limit the cell-cell interactions and subsequently decreased the chondrogenic differentiation [43]. In another study, Zhou et al. synthesized three-dimensional (3D) printed GO scaffold by incorporating GO, gelatin methacrylate (GelMA), and poly (ethylene glycol) diacrylate (PEGDA) for promoting chondrogenic differentiation of hMSCs. The GelMA-PEGDA-GO scaffolds could significantly elevate the chondrogenic gene expression including, type II collagen, SOX-9, and aggrecan [44].

Several studies have found that under appropriate culture conditions MSCs could also differentiate into non-mesodermal lineages, including neural cells. Because neural cells are an electroactive tissue that responds to the electrical stimuli, an electrically conductive scaffold could be used to promote neural differentiation. However, the oxygen functional groups of GO could disrupt the electronic structure of graphene. Therefore, a reduction of the oxygen functional groups is necessary in order to recover the electrical conductivity of graphene structure. The combination between electrical stimulation and graphene was synergistically effective on neurogenesis. Recently, there are some studies that utilized rGO-based nanomaterials for promoting neural differentiation of MSCs. Lim et al. observed the combination between pulsed electromagnetic field (PEMF) as an electrical stimulation and rGO to investigate the neurogenesis of MSCs. The study showed that rGO and PEMF could significantly enhance the expression of Nestin and Microtubule-associated protein 2 (MAP2), which are the important neurogenic differentiation markers [45]. Furthermore, rGO could be functionalized with other materials in order to enhance the neurogenesis more. Guo et al. combined the electrical stimulation signals by using triboelectric nanogenerator (TENG) and poly(3,4-ethylenedioxythiophene) (PEDOT)-rGO hybrid microfiber (80 μm in diameter) as a conductive scaffold for promoting neural differentiation of MSCs. The experiments showed that the addition of PEDOT could enhance the Neuron-specific Class III β-tubulin (Tuj1) expression (early neurogenic differentiation marker) by ∼1.68-fold over that on the rGO microfiber [29].

### 3.4. Effect on Neural Stem Cell Differentiation

Neural stem cells (NSCs) are self-renewing and multipotent cells that are located in small areas of the brain, called subgranular zone (SGZ) and subventricular zone (SVZ) [46,47]. NSCs have the ability to differentiate into neurons, astrocytes, and oligodendrocytes [48]. The stimulation of NSC differentiation is very important for the application in neural tissue engineering and neural regeneration. It is well known that nerve is an electro-active tissue that responds to the electrical stimuli. Therefore, the utilization of electrical fields (EFs) is recently being established to guide neurogenesis through a stem cell differentiation process [49,50].

There are two sources of electrical stimulation, such as endogenous and exogenous electrical fields (EFs). Endogenous EFs are bioelectric fields that produced by the cells across their plasma membrane in which very essential for maintaining cellular homeostasis and are evoked in many biological events. On the other hand, exogenous EFs are bioelectric fields that generated from external power sources and are generally applied to biological cells/tissues via electrodes [51]. In recent years, the utilization of nanomaterials that have electro-conductivity properties to transmit the electric signals from EFs have attracted the field of neuroscience, especially in NSC differentiation research. Due to its remarkable electro-conductivity, rGO has been utilized as a controller of NSC differentiation to neuronal lineage.

Recent studies have shown that rGO, both as a single and hybrid materials, can potentially promote NSC differentiation towards neurons, which is characterized by the elevation of Tuj1 and MAP2 expression levels. Comparing to GO, rGO has better electro-conductivity properties due to the removal most of its oxygen groups that subsequently recovering the electrical conductivity of graphene structure [17,52]. However, there are several reports showed that GO could also enhances the differentiation of NSCs and even better than rGO, which might be due to the surface hydrophilicity of GO [16,53]. The additional exogenous electrical stimulation often combined with GO and rGO to improve the differentiation efficiency [29,54]. As conductive materials, GO and rGO lend themselves for a more efficient delivery of the electrical stimulation. Nevertheless, without the help from exogenous EFs, GO and rGO can still promote the NSC differentiation. It is due to the electro-conductivity property of GO and rGO, which can conduct the endogenous EFs into NSCs, and subsequently stimulate the differentiation of NSCs.

### 3.5. Effect on Cancer Stem Cell Differentiation

Cancer stem cells (CSCs) are the subpopulation of tumor cells that have the ability to self-renew, differentiate, and form new tumor cells. CSCs are responsible for the tumor initiation, growth, metastasis, and relapse. Moreover, CSCs are resistant to the conventional therapy, such as chemotherapy, due to its drug resistance ability by releasing various agents to the extracellular environment via efflux mechanism by ATP-binding cassette (ABC) transporter [55]. CSCs have the same differentiation ability like normal stem cells. Therefore, promoting the differentiation of CSC could be an effective approach. 

Accumulating evidences have showed that CSCs could differentiate into various cancer cells, including colon, pancreatic, liver, prostate, blood, and lung cancers [56,57,58,59,60]. In addition, CSCs also have the ability to transdifferentiate into normal non-cancer cells, such as vascular endothelial cells and pericytes [61,62,63]. There is evidence that GO also plays an important roles in the differentiation of CSC. Cancer stem cells differentiate to form a small mass of cells known as a tumor-sphere. The GO flakes prevented CSCs from forming that tumor-sphere, and instead forced them to differentiate into non-cancer stem-cells. The signal transduction cascades, including Wnt, Notch, and Hedgehog signaling pathways that are very important for CSC self-renewal are suppressed by GO. This report concluded that GO could possibly be an effective therapeutic strategy to eradicate CSCs via differentiation-based therapy [64].

## 4. Possible Underlying Mechanisms of Graphene Oxide and Stem Cell Interaction

**Figure 1:**
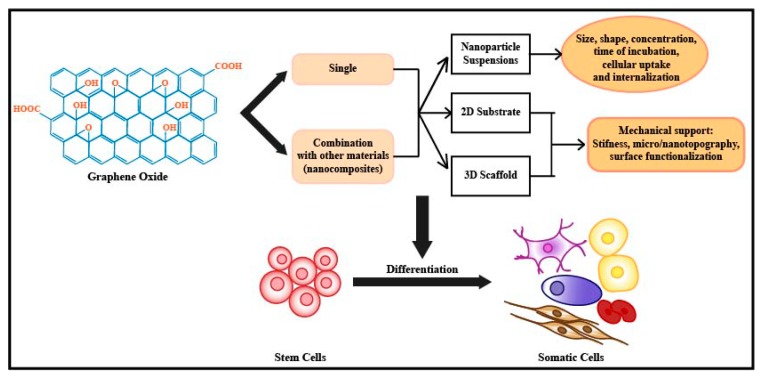
Schematic diagram depicting the guidance of stem cell differentiation using graphene oxide-based nanomaterials.

With the increasing interest in graphene oxide, a number of studies have tried to investigate the interaction between GO and various types of stem cells in order to elucidate the underlying mechanism of toxicity and differentiation. Several papers have shown that GO-based nanomaterials could be used in the form of nanoparticles suspension, 2D substrate, and 3D scaffold for modulating stem cell growth and differentiation (Figure 1). As described above, the interaction between GO and stem cells is highly influenced by several parameters, including size, shape, concentration, time of incubation, and surface chemistry. Moreover, GO in the forms of 2D substrates and 3D scaffolds have additional parameters that could influence the stem cell behaviors, such as substrate stiffness, conductivity, and topography (Table 2). 

GO in the form of nanoparticles suspension could be used as a drug carrier and stimulator for promoting stem cell differentiation [23,37,65]. The most important parameter that influences the GO in the form of nanoparticles suspension is the intracellular localization. Several papers have been reported that nanoparticles could easily pass the cell membrane and translocate into the cytoplasm or nucleus and subsequently alter the cellular signaling pathways. However, the intracellular localization of nanoparticles is strongly influenced by the particles size. For example, GO at the microscale size could not pass the cell membrane and it would only adhere at the cell membrane [66]. Whereas, GO at the smaller size (400 nm), some of them could be localized in cytoplasm. However, in that study, the authors concluded that the presence of GO in the cytoplasm did not have any significant roles due to its low concentration [27]. Therefore, the concentration of the nanoparticles suspension also has a critical role in regulating stem cell differentiation (Table 2). In addition, the cellular pathways may differ depending on the stem cell types. Wei et al, reported that pristine GO nanosheets could promote the osteogenic differentiation of MSCs via the activation of Wnt/β-catenin signaling pathway [23]. On the other hand, Fiorillo et al found that GO (big and small flakes) could inhibit the signaling transduction pathways that contribute for the stemness of CSC, including Wnt, STAT3, Notch, and Nuclear factor-like 2 (Nrf2), resulting in the enhancement of CSC differentiation. Furthermore, there is another study that reported GO nanosheets could maintain the stemness of ESC via downregulating the downstream genes of integrin signaling, including phospho-focal adhesion kinase (p-FAK), Vinculin, and Ras-related protein 1 (Rap1) [64].

Accumulating evidences have shown that biophysical cues, such as surface stiffness and nanotopography, could affect the stem cell growth and differentiation [69,70,71]. Recently, the application of GO as 2D substrates and 3D scaffold on promoting stem cell growth and differentiation have been extensively studied due to their mechanical supports, including stiffness, conductivity, surface chemistry, and topography. Kang et al. have demonstrated that GO flakes could increase the mechanical stiffness of collagen sponge scaffold and subsequently enhanced the osteogenic differentiation of MSCs. Furthermore, they have also showed the elevated osteogenic differentiation is mediated by intracellular mechanotransduction pathways. The protein expressions of FAK and Vinculin, which have important roles in response to substrate environment were elevated and the downstream signaling pathway, Extracellular signal-regulated kinase (ERK), was also activated to transfer the mechanical information into the cell nucleus, which further enhancing the osteogenic differentiation [26]. The electrical conductivity of GO also plays a critical role in differentiating electo-active tissue, including neurons and myoblast. Chaudhuri et al. have showed the importance of GO conductivity on promoting myoblast differentiation of human MSCs. The incorporation of GO into polymer composite meshes could enhance the conductivity of the substrate and provided supporting cues to stimulate the myotubes formation [72]. Yang et al. demonstrated that GO-based patterned substrates could be an effective culture platform for inducing neuronal differentiation of NSCs by activating focal adhesion signaling via phosphorylation of focal adhesion kinase and paxilin [73]. In addition, it is important to highlight that the influence of GO on the proliferation and differentiation of stem cells also comes from the active interactions of GO with key biomolecules, such as nucleic acids, proteins, and ATP via supramolecular binding. It has been found that single-stranded nucleic acids bind to GO and they can be transported through the cell membrane [74,75]. Furthermore, due to the oxygen-functional groups of GO, the hydrophilic domain of GO could bind to serum proteins via electrostatic interactions, which lead to the enhancement of cell adhesion and growth [40]. The supramolecular ensembles of GO with ATP have also been described recently [76]. The ability of GO to interact with ATP indicates that GO can influence the energy production and transfer. Thus, influencing the life processes and signaling pathways.

## 5. Conclusions and Future Directions

As described in this mini review, GO has shown excellent performance in regulating the self-renewal and differentiation processes of stem cells. GO could be applied in three different forms, including nanoparticles suspension, 2D substrates, and 3D scaffolds to regulate stem cell growth and differentiation with a different mechanism. However, there are still some challenges that need to be overcome in order to use GO-based nanomaterial for clinical application. First, more studies are still needed to dissect the exact molecular mechanisms in which GO contributes to the stem cell growth and differentiation. Second, the in vivo study must be conducted to ensure the effects of GO-based nanomaterial. Lastly, study about the combination of GO with other biomaterials to form 3D nano-hybrid composites scaffold should be advanced.

## Figures and Tables

**Table 1:** Biocompatibility of graphene oxide (GO)-based nanomaterials in stem cells.

Material	Stem Cell Type	Parameter Studied	Results	References
GO	MSCs	Concentration	Safe dose: ≤0.1 µg/mL	[23]
rGO	MSCs	Concentration and size	Cytotoxicity: Small rGO > Large rGO Safe dose: ≤1 µg/mL (small rGO) ≤100 µg/mL (large rGO)	[24]
GNO, GONR, GONP	MSCs	Concentration, time of incubation, and shape	Viability: GNO > GONR > GONP Safe dose: ≤50 µg/mL There is no effect of incubation time on cell viability	[25]
GO	ESCs	Concentration	Safe dose: ≤32 µg/mL	[26]
GO	NSCs	Concentration	Safe dose: ≤5 µg/mL	[27]
ADM, ADM-GO/Que, and ADM-GO-PEG/Que	MSCs	Surface functionalization	Proliferation: ADM-GO-PEG/Que > ADM-GO/Que > ADM	[28]
rGO/PEDOT	MSCs	Surface functionalization	Proliferation: rGO/PEDOT > rGO	[29]
PCL/GO, PCL/rGO, PCL/AGO	MSCs	Surface functionalization	Proliferation: PCL/AGO > GO > rGO	[22]

**Notes:** ADM: Acellular dermal matrix; AGO: Amine-functionalized graphene oxide; ESCs: embryonic stem cells; GNO: Graphene oxide nano-onions; GO: graphene oxide; GONP: Graphene oxide nanoplatelets; GONR: Graphene oxide nanoribbons; MSCs: Mesenchymal stem cells; NSCs: Neural stem cells; PCL: poly(ε-caprolactone); PEDOT: poly(3,4-ethylenedioxythiophene); PEG: polyethylene glycol; Que: Quercetin; rGO: reduced graphene oxide.

**Table 2:** Summary of the application of GO-based nanomaterial in stem cell differentiation.

Material	Surface Modification	Stem Cell Type	Differentiation	Parameters that Influence the Differentiation	References
*Nanoparticle suspensions*					
CNTs, GO, Gp		ESCs	Dopamine neuron	Concentration	[32]
GO nanosheets		MSCs	Osteogenic	Concentration	[23]
GO		NSCs	Neurogenic	Concentration	[27]
GO		CSCs	Non-CSCs	Size and concentration	[64]
Gp, GO, and porous GO		MSCs	Chondrogenic	Concentration	[43]
GO		MSCs	Osteogenic and adipogenic	Concentration, incubation time, and shape.	[25]
*2D substrates*					
GO		ESCs	Haematopoetic		[31]
GO and Gp		iPSCs	Endoderm	Surface chemistry	[34]
GO and Gp		MSCs	Osteogenic and adipogenic	Surface chemistry	[40]
GO		MSCs	Osteogenic	Size	[67]
GO	Methacrylate	MSCs	Osteogenic	Nanotopography and surface chemistry	[38]
rGO	Microfiber	NSCs	Neurogenic	Surface topography	[52]
GO and rGO		ADSCs	Neurogenic	Surface chemistry	[16]
*3D scaffolds*					
GO	Collagen sponge	MSCs	Osteogenic	Stiffness	[39]
GO foam	Surface rolled	NSCs	Neurogenic	Electrical conductivity	[54]
GO	PEG and Quercetin	MSCs	Osteogenic and adipogenic	Surface topography	[28]
GO	Polypeptide thermogel	MSCs	Adipogenic	Surface chemistry and stiffness	[41]
GO	PLGA nanofiber	MSCs	Osteogenic	Surface chemistry	[68]
GO	GELMA and PEGDA	MSCs	Chondrogenic	Surface chemistry and stiffness	[44]

**Notes:** 2D: 2 dimension; 3D: 3 dimension; ADSCs: adipose-derived stem cells; CNTs: carbon nanotubes; CSCs: Cancer stem cells; ESCs: embryonic stem cells; GELMA: gelatin methacrylate; GO: graphene oxide; Gp: graphene; iPSCs: induced pluripotent stem cells; MSCs: Mesenchymal stem cells; NSCs: Neural stem cells; PEG: polyethylene glycol; PEGDA: polyethylene glycol diacrylate; PLGA: poly(lactic-co-glycolic acid); rGO: reduced graphene oxide.
